# Establishing a predicted model to evaluate prognosis for initially diagnosed metastatic Her2-positive breast cancer patients and exploring the benefit from local surgery

**DOI:** 10.1371/journal.pone.0242155

**Published:** 2020-11-10

**Authors:** Hong Lin, Yanxuan Wu, Guoxi Liang, Liming Chen

**Affiliations:** 1 Department of Oncology, the First Affiliated Hospital of Shantou University Medical College, Shantou, Guangdong, China; 2 Shantou University Medical College, Shantou, Guangdong, China; 3 Department of Radiation Oncology, Cancer Hospital of Shantou University Medical College, Shantou, Guangdong, China; Universita degli Studi di Torino, ITALY

## Abstract

**Background:**

For patients initially diagnosed with metastatic Her2-positive breast cancer (MHBC), we intended to construct a nomogram with risk stratification to predict prognosis and to explore the role of local surgery.

**Methods:**

We retrieved data from the Surveillance, Epidemiology, and End Results (SEER) database. Kaplan–Meier (KM) method and log-rank test were used for the selection of significant variables. Cox regression analysis and Fine-Gray test were utilized to confirm independent prognostic factors of overall survival (OS) and breast cancer-specific survival (BCSS). A nomogram predicting 1-year, 3-year, and 5-year OS was developed and validated. Patients were stratified based on the optimal cut-off values of total personal score. KM method and log-rank test were used to estimate OS prognosis and benefit from local surgery and chemotherapy.

**Results:**

There were 1680 and 717 patients in the training and validation cohort. Age, race, marriage, T stage, estrogen receptor (ER) status, visceral metastasis (bone, brain, liver and lung) were identified as independent prognostic factors for OS and BCSS, while histology was also corelated with OS. C-indexes in the training and validation cohort were 0.70 and 0.68, respectively. Calibration plots indicated precise predictive ability. The total population was divided into low- (<141 points), intermediate- (142–208 points), and high-risk (>208 points) prognostic groups. Local surgery and chemotherapy brought various degrees of survival benefit for patients with diverse-risk prognosis.

**Conclusions:**

We constructed a model with accurate prediction and discrimination. It would provide a reference for clinicians' decision-making. Surgery on the primary lesion was recommended for patients with good physical performance status, while further study on optimal surgical opportunity was needed.

## Introduction

Breast cancer (BC) is the most leading malignancy among females worldwide, with an estimation of 600,000 deaths of women in 2017 [1]. Recent statistics demonstrated that approximately 5–8% of breast neoplasms at initial diagnosis are metastatic breast cancers (MBC), which are conferred a worse prognosis and a period of narrower survival time [2]. Additionally, 20–30% of BC patients suffer from distant metastases upon initial diagnosis and treatment, and the median survival time of MBC patients is 2–3 years [3, 4]. For a long time, tumor burden and metastasis pattern have been identified as prognostic factors in MBC patients [5]. Bone, liver, lung, and brain are the most common regions of visceral metastases, and the prognosis of patients with brain metastases is worse than that of patients with other oligometastasis [6, 7]. Meanwhile, the clinical outcomes of MBC patients are quite different due to the molecular subtype, tumor grade, hormone receptor, and other biologic characteristics [8]. Of important, Smid et al. [9] substantiated that molecular typing relied on the expression of estrogen receptor (ER), progesterone receptor (PR), and human epidermal growth factor receptor 2 (Her2), which had important implications for the metastatic pattern and prognosis of advanced BC. Her2-positive status generally indicates a poor prognosis. It is obviously associated with risk of early relapse and is more prone to undergo visceral metastases [10, 11]. Of course, Her2-positive status also has relatively positive effects, such as higher pathologic complete response (pCR) after targeted-neoadjuvant therapy [12]. Survival rate of patients initially diagnosed with metastatic Her2-positive breast cancer (MHBC) varies widely because of the clinical heterogeneity [8, 10, 13].

With the recommendation of incorporating molecular subtypes to the prognosis assessment of BC in the American Joint Committee on Cancer (AJCC) 8th edition cancer staging, traditional TNM staging has been unable to meet the basic clinical need for various BC patients [14]. A precise prediction model contains clinical and biologic features is urgently needed to evaluate the prognosis of patients with initially diagnosed MHBC, and it could be potentially beneficial to select appropriate treatment and arrange disease monitoring for clinicians.

Nomogram is a graphical calculation model with personalized risk prediction efficiency. It can quantify the risk of clinical events by incorporating all independent prognostic factors for statistical calculations [15, 16]. We verified the parameters that affected the overall survival (OS) of initially diagnosed MHBC patients and quantified these parameters via establishing nomogram, so we can predict the prognosis and provide optimal therapeutic strategies for these patients. The model can be used as a practical auxiliary tool for clinicians.

## Materials and methods

### Population

Information of patients diagnosed as BC by positive histopathology between 2010 and 2016 was collected from the Surveillance, Epidemiology, and End Results (SEER) 18 registries. We used the SEER program statistical analysis software package (SEER*Stat 8.3.6, available at https://seer.cancer.gov/seerstat/) to identify patients. The inclusion criteria were: (1) patients were diagnosed with MHBC; (2) female patients were no more than 70 years old; (3) BC was the only primary malignancy; (4) patients were more than 20 years old; (5) patients had more than 1-month survival time; (6) patients with unilateral malignancy. Otherwise, patients with incomplete imperative information were excluded.

As personal identifying information was not covered in our study. The ethic application to the Institutional Review Board was waived. The study protocol conforms to the provisions of the Helsinki Declaration as revised in 2013.

### Demographic and clinicopathological information

Baseline features included follow-up time, survival status, cause of death, age at diagnosis, the race of patients, marriage status, pathological differentiation, histology, T staging, N staging, visceral metastasis status (bone, brain, liver, lung), ER status, PR status, surgery on primary lesion, and chemotherapy record were extracted from SEER database. The marriage status of patients including married, unmarried and other (including divorced, windowed, separated, and domestic partner patients). In addition, we divided histologic types into 3 subgroups, including infiltrating ductal carcinoma (IDC), infiltrating lobular carcinoma (ILC) and other histologic type. In addition, as a continuous variable, we transformed the age at diagnosis into a categorical parameter to meet the need of nomogram construction.

### Statistical analyses

Patients extracted from the SEER database were randomly classified into the training and validation cohort in a ratio of 7:3. The training cohort was utilized to develop a nomogram for predicting 1-year, 3-year, 5-year OS probability of initially diagnosed MHBC patients and substantiate the independent prognostic factors for breast cancer-specific survival (BCSS). It was also applied to internal validation, while external validation was implemented in the validation cohort.

OS was defined as the survival duration by the time of BC diagnosis to death, or to the last recorded follow-up for patients alive. We utilized the Kaplan–Meier (KM) method and log-rank test for each parameter to identify univariate prognostic factors. Significant variables were incorporated into the Cox proportional hazards regression. Independent prognostic factors contributed to OS were notarized and applied to construct nomogram. A score of 0–100 was assigned to each subgroup of independent prognostic factors based on the regression coefficient obtained via Cox survival analysis. An indicator with the greatest influence on OS was conferred with a score of 100. Scores of other indicators were converted according to the relative proportion of regression coefficient. Finally, the score of each independent prognostic factor was applied to construct nomogram. This process was implemented by using the ‘rms’ package via R. The validation cohort was used to verify the predictive model. We employed Harrell’s C statistic concordance index (C-index) and calibration plots to estimate discrimination and accuracy of the nomogram. Conventionally, the value of C-index ranges from 0.5–1.0, with 0.5 represents a random choice while 1.0 indicates perfect discrimination. Calibration plots evaluated the consistency between the predicted and actual survival probability by comparing the calibration line with an optimal 45-degree line. C-index and calibration plots were applied in both internal and external validation. Bootstrap with 1,000 reiterations was used for these analyses.

BCSS was defined as the survival time from the time of BC diagnosis to the death caused by BC, whereas the death caused by other events was regarded as competitive events. We used Fine-Gray test to perform univariate and multivariate analyses in the training cohort, in order to identify the independent prognostic factors contributed to BCSS for MHBC patients [17].

According to the nomogram, we calculated the total personal score for all patients. We assigned the total cohorts into three different prognostic groups by utilizing the optimal cut-off of personal score which was obtained from X-title software [18]. KM curve was established to assess OS probability for diverse-risk prognostic patients. Cumulative incidence function (CIF) curves were constructed to estimate breast cancer-specific mortality and competitive mortality of diverse-risk prognostic patients. Additionally, the benefit from local surgery and chemotherapy in different prognostic groups was evaluated by using the KM method and log-rank test.

Statistical analyses in our retrospective research were completed by using X-title, SPSS (version 23.0) and R (version 3.6.3). Two-side p-value < 0.1 was considered statistically significant in univariate analysis, while that < 0.05 was considered significant in other analyses.

## Results

### Baseline characteristics of the population

**Fig 1:**
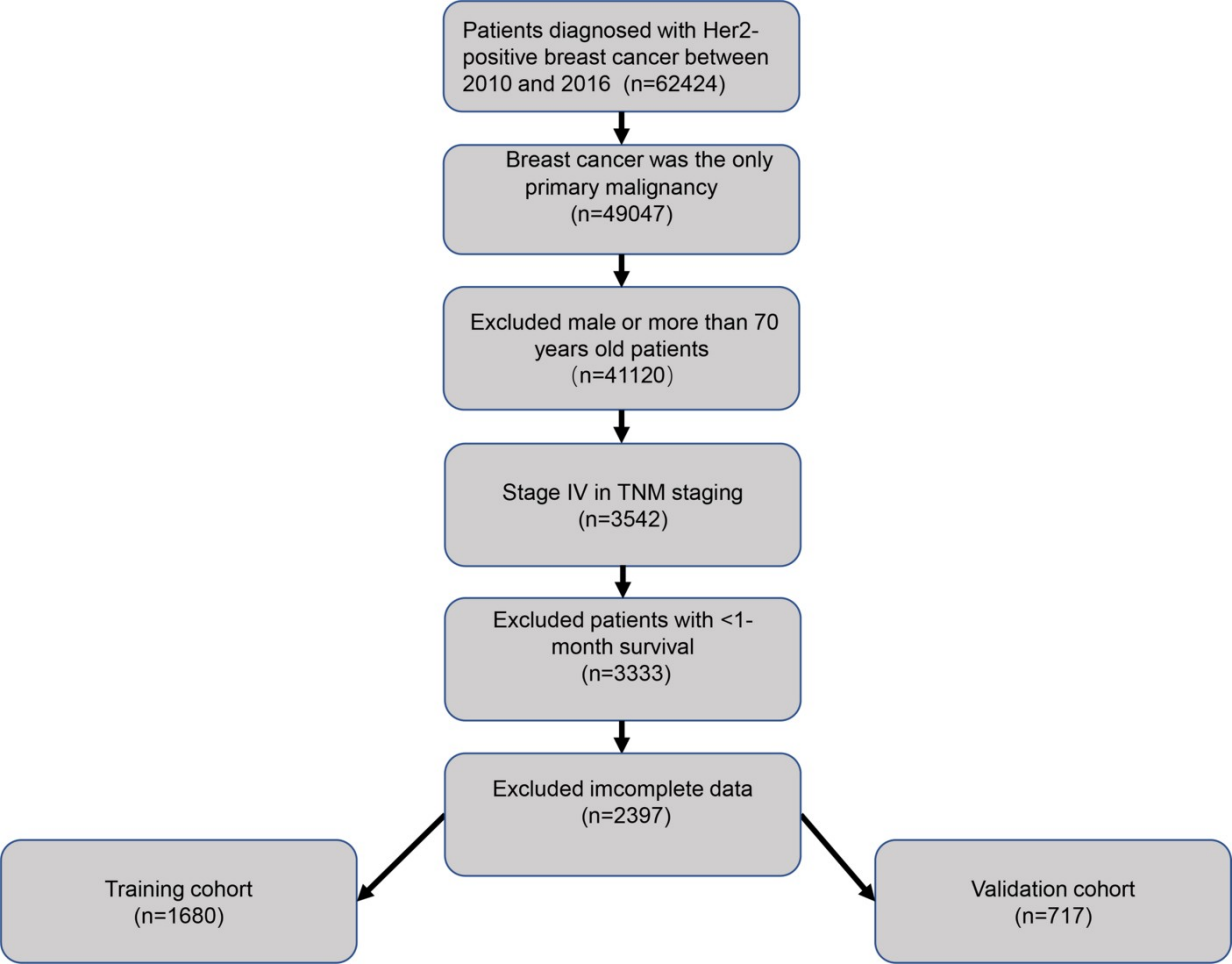
The flowchart of data extraction; Her 2, human epidermal growth factor receptor 2.

As showed in Fig 1, a total of 2397 patients from the SEER database were enrolled in our retrospective study. Demographics features and clinicopathologic characteristics of the population were listed in Table 1. The median age of all patients was 54 (IQR, 45–61) years old and median follow-up time was 23 (IQR, 9–41) months. Total cohorts were randomly classified into the training and validation cohort, of which 1680 in the training cohort while 717 in the validation cohort. In the training cohort, 597 (35.5%) cases were reported death, among which 562 (94.1%) were due to breast cancer-specific events. Analogously, 236 (32.9%) patients in the validation cohort were reported, among which 218 (92.4%) were caused by breast cancer-specific events.

**Table 1:** Demographics futures and clinicopathologic characteristics of including patients.

Variables	Training cohort (n = 1680)		Validation cohort (n = 717)		p value
	Number	Percentage (%)	Number	Percentage (%)	(chi-square test)
**Age**					0.876
≤40y	274	0.16	111	0.15	
41-55y	683	0.41	293	0.41	
56-70y	723	0.43	313	0.44	
**Race**					0.266
White/Asian/AI	1379	0.82	602	0.84	
Black	301	0.18	115	0.16	
**Marriage**					0.637
Married	889	0.53	375	0.52	
Unmarried	444	0.26	182	0.25	
Other*	347	0.21	160	0.22	
**Pathological grade**					0.218
Well/moderately differentiated	563	0.34	259	0.36	
Poorly/un- differentiated	1117	0.66	458	0.64	
**Histology**					0.152
IDC	1461	0.87	636	0.89	
ILC	54	0.03	13	0.02	
Other	165	0.10	68	0.09	
**T stage**					0.914
T4	633	0.38	266	0.37	
T3	320	0.19	134	0.19	
T2	549	0.33	234	0.33	
T0-1	178	0.11	83	0.12	
**Lymph node metastasis**					0.831
Positive	1407	0.84	603	0.84	
Negative	273	0.16	114	0.16	
**Bone metastasis**					0.356
Yes	964	0.57	426	0.59	
No	716	0.43	291	0.41	
**Brain metastasis**					0.793
Yes	127	0.08	52	0.07	
No	1553	0.92	665	0.93	
**Liver metastasis**					0.054
Yes	621	0.37	295	0.41	
No	1059	0.63	422	0.59	
**Lung metastasis**					0.943
Yes	513	0.31	220	0.31	
No	1167	0.69	497	0.69	
**ER status**					0.513
Positive	1064	0.63	444	0.62	
Negative/borderline	616	0.37	273	0.38	
**PR status**					0.613
Positive	752	0.45	329	0.46	
Negative/borderline	928	0.55	388	0.54	
**Surgery**					0.793
Yes	715	0.43	301	0.42	
No	965	0.57	416	0.58	
**Chemotherapy**					0.042
Yes	1446	0.86	639	0.89	
No	234	0.14	78	0.11	
**Survival status**					0.217
Alive	1083	0.64	481	0.67	
Dead	597	0.36	236	0.33	

*Including divorced, windowed, separated, and domestic partner patients; AI, American Indian race.

IDC, infiltrating ductal carcinoma; ILC, infiltrating lobular carcinoma; ER: Estrogen receptor; PR, progesterone receptor.

In terms of visceral metastasis, bone, brain, liver and lung metastasis accounted for 58.0% (1390/2397), 7.5% (179/2397), 38.2% (916/2397), 30.6% (733/2397) of the total population, respectively. Bone metastasis was the most common visceral metastasis, while brain metastasis was the least common one. The result from both KM method and log-rank test implied that all 4 types of visceral metastasis were significantly related to a worse prognosis (p<0.001). Median survival time for patients with brain, bone, liver, lung metastases was 12 (IQR, 4–28), 21 (IQR, 8–39), 21 (IQR, 8–38), 19 (IQR, 8–34) months, respectively. The prognosis of patients with brain metastasis was worst, while patients with bone, lung and liver metastasis had a semblable prognosis. As for hormone receptors status, there were 1508 (62.9%) patients with positive ER, while 1081 (45.1%) patients with positive PR. There were 1016 (42.4%) patients underwent surgery on the primary lesion after a BC diagnosis.

### Parameters associated with OS and BCSS

In the training cohort, we performed univariate and multivariate analyses to verify independent prognostic factors contributed to OS and BCSS. Age at diagnosis, the race of patients, marital status, histology, T stage, lymph node metastasis, bone metastasis, brain metastasis, liver metastasis, lung metastasis, ER status and PR status were theoretically correlated with OS of MHBC patients by univariate analysis. Further multivariate analysis confirmed that aforementioned variables except lymph node metastasis and PR status were independent prognostic factors for OS (p<0.05). Brain metastasis brought the greatest threat to OS probability (HR 3.13, 95%CI 2.47–3.97, p<0.001), and patients with venerable age was also considered to have a relatively worse prognosis (for patients with 56–70 years old, HR 2.30, 95%CI 1.72–3.07, p<0.001). Patients with bone, liver and lung metastasis would increase a 44%, 53%, 46% of all-cause mortality, respectively. Patients with positive ER status generally obtained a prolonged survival duration (HR 1.43, 95%CI 1.21–1.69, p<0.001).

Furthermore, age, race, marriage status, T stage, bone metastasis, brain metastasis, liver metastasis, lung metastasis, ER status were independent prognostic factors for BCSS of MHBC patients via Fine-Gray univariate and multivariate analyses. The results of univariate and multivariate analyses for OS and BCSS were shown in Table 2.

**Table 2:** Univariate and multivariate analyses of OS and BCSS in the training cohort.

	OS			BCSS		
Variables	Univariate analysis	Multivariate analysis		Univariate analysis	Multivariate analysis	
	p value	HR (95% CI)	p value	p value	HR (95% CI)	p value
**Age**	<0.001			<0.001		
≤40y		Reference			Reference	
41-55y		1.82(1.37,2.44)	<0.001		1.82(1.36,2.44)	<0.001
56-70y		2.30(1.72,3.07)	<0.001		2.29(1.71,3.06)	<0.001
**Race**	<0.001			<0.001		
White/Asian/AI		Reference			Reference	
Black		1.50(1.23,1.83)	<0.001		1.34(1.06,1.69)	<0.001
**Marriage**	0.04			<0.001		
Married		Reference			Reference	
Unmarried		1.22(1.00,1.49)	0.050		1.31(1.07,1.60)	0.01
Other*		1.24(1.01,1.51)	0.042		1.23(0.98,1.53)	0.07
**Histology**	0.035			0.056		
IDC		Reference			Reference	
ILC		1.46(0.94,2.26)	0.089		1.49(0.96,2.32)	0.074
Other		1.37(1.07,1.77)	0.014		1.29(0.98,1.68)	0.066
**Differentiated**	0.514			0.408		
**T stage**	<0.001			<0.001		
T4		Reference			Reference	
T3		0.88(0.70,1.11)	0.285		0.95(0.76,1.19)	0.660
T2		0.77(0.63,0.94)	0.010		0.77(0.62,0.95)	0.014
T0-1		0.78(0.58,1.04)	0.087		0.71(0.52,0.96)	0.028
**Lymph node metastasis**	0.096			0.495		
Positive		Reference				
Negative			0.565			
**Bone Metastasis**	<0.001			<0.001		
Yes		1.44(1.21,1.70)	<0.001		1.39(1.17,1.66)	<0.001
No		Reference			Reference	
**Brain Metastasis**	<0.001			<0.001		
Yes		3.13(2.47,3.97)	<0.001		3.05(2.33,3.99)	<0.001
No		Reference			Reference	
**Liver Metastasis**	<0.001			<0.001		
Yes		1.53(1.30,1.80)	<0.001		1.52(1.28,1.81)	<0.001
No		Reference			Reference	
**Lung Metastasis**	<0.001			<0.001		
Yes		1.46(1.22,1.73)	<0.001		1.46(1.21,1.76)	<0.001
No		Reference			Reference	
**ER status**	0.006			<0.001		
Positive		Reference			Reference	
Negative/borderline		1.43(1.21,1.69)	<0.001		1.28(1.03,1.59)	0.028
**PR status**	0.002			<0.001		
Positive		Reference			Reference	
Negative/borderline			0.422			0.330

OS, overall survival; BCSS, breast cancer-specific survival; HR, hazard ratio; CI, confidence intervals.

*Including divorced, windowed, separated, and domestic partner patients; AI, American Indian race.

IDC, infiltrating ductal carcinoma; ILC, infiltrating lobular carcinoma; ER: Estrogen receptor; PR, progesterone receptor.

### Nomogram construction and validation

**Fig 2:**
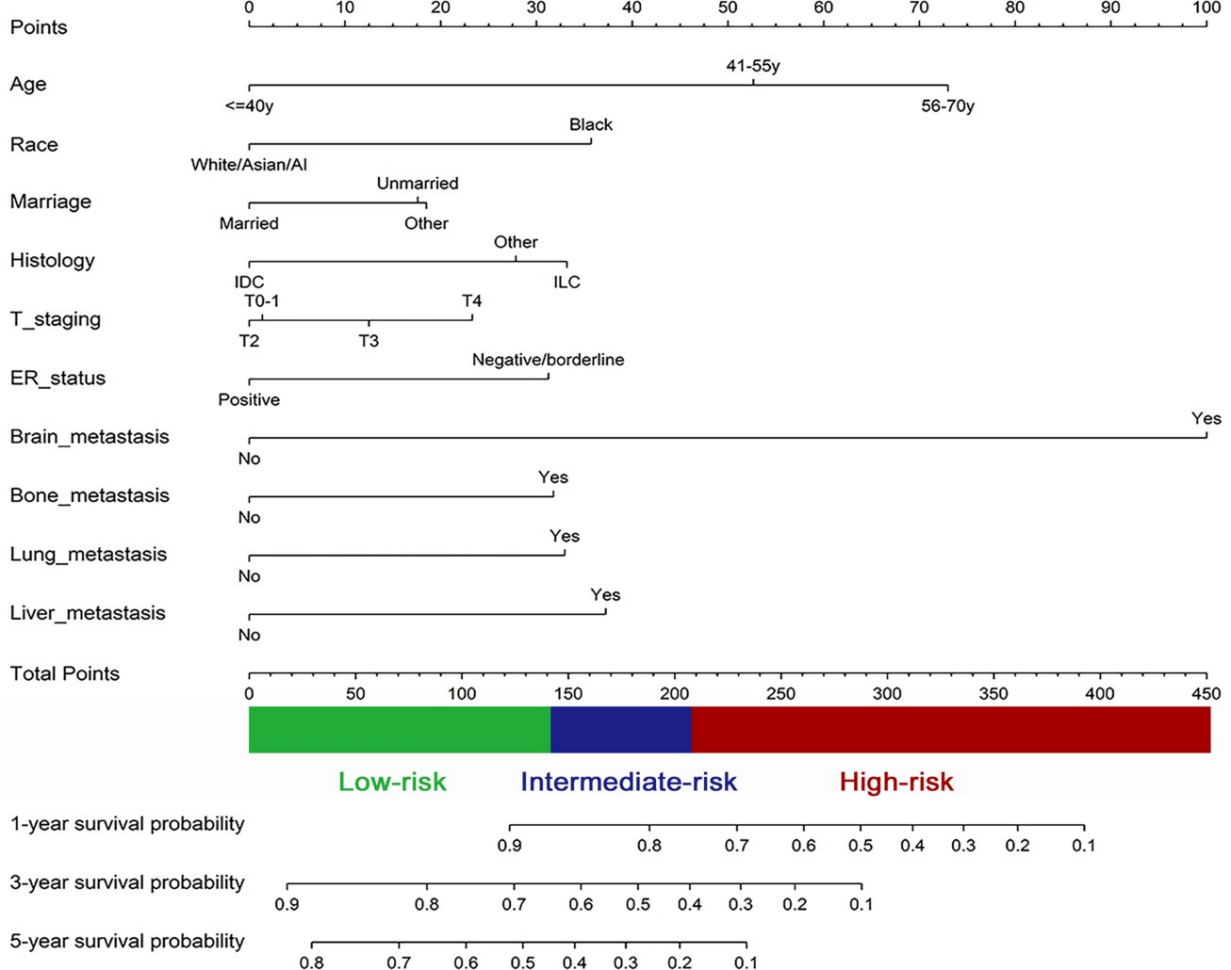
The nomogram for predicting 1-year, 3-year, 5-year overall survival probability of metastatic Her2-positive breast cancer patients; IDC, infiltrating ductal carcinoma; ILC, infiltrating lobular carcinoma; ER: Estrogen receptor.

Ten independent prognostic factors were applied to generate a nomogram for predicting 1-year, 3-year, 5-year OS probability for initially diagnosed MHBC patients in the training cohort (Fig 2). Table 3 showed coefficients and scores of 10 prognostic factors. Brain metastasis brought the worst prognosis for MHBC patients and it was conferred 100 points. For other prognostic factors, subgroup with the least prognostic risk was conferred 0 point. This nomogram will be used to quickly predict patients’ prognosis. When a patient is initially diagnosed as MHBC, clinicians obtain corresponding score according to the clinicopathological data of this patient, sum up each score, locate the total points on ‘total points’ axis and draw a vertical line extending to the “1-/3-/5-year survival probability” axis to obtain OS probability.

**Table 3:** Coefficient and score for each subgroup of all variables.

Variables	Coefficient	Points	Variables	Coefficient	Points
Age			T stage		
≤40y	-	0	T4	0.26	23
41-55y	0.60	53	T3	0.15	13
56-70y	0.83	73	T2	-	0
Race			T0-1	0.01	1
White/Asian/AI	-	0	Bone metastasis		
Black	0.41	36	No	-	0
Marriage			Yes	0.36	32
Married	-	0	Brain metastasis		
Unmarried	0.20	18	No	-	0
Other*	0.21	19	Yes	1.14	100
Histology			Liver metastasis		
IDC	-	0	No	-	0
ILC	0.38	33	Yes	0.43	37
Other	0.32	28	Lung metastasis		
ER status			No	-	0
Positive	-	0	Yes	0.38	33
Negative/borderline	0.36	31			

*Including divorced, windowed, separated, and domestic partner patients; AI, American Indian race.

IDC, infiltrating ductal carcinoma; ILC, infiltrating lobular carcinoma; ER: Estrogen receptor.

**Fig 3:**
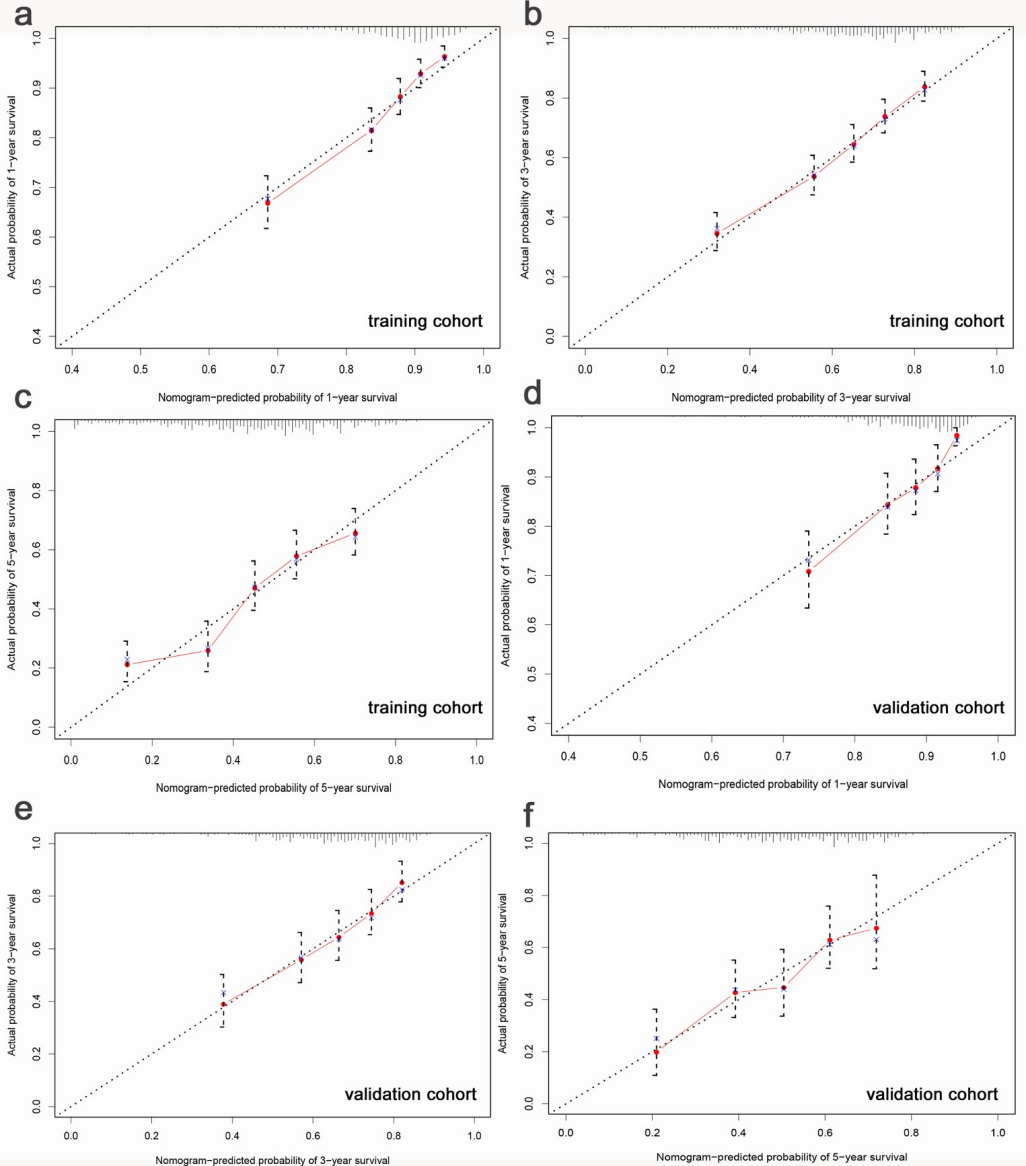
Calibration plots of the nomogram for predicting 1-year, 3-year, 5-year overall survival probability of metastatic Her2-positive breast cancer patients in the training (a-c) and validation cohort (d-f). All the 6 Calibration plots showed great consistency between the predicted lines and 45-degree lines.

The C-indexes for the nomogram were 0.70 (95% CI, 0.68–0.72) and 0.68 (95% CI, 0.65–0.72) in the training and validation cohort, respectively, which implied the favor discrimination of the model. Calibration plots were established to evaluate the predicted accuracy of the nomogram (Fig 3). The predicted survival probability basically conformed the actual survival probability in both internal and external validation, which ascertained well exactitude of our predicted model.

### Risk stratification

**Fig 4:**
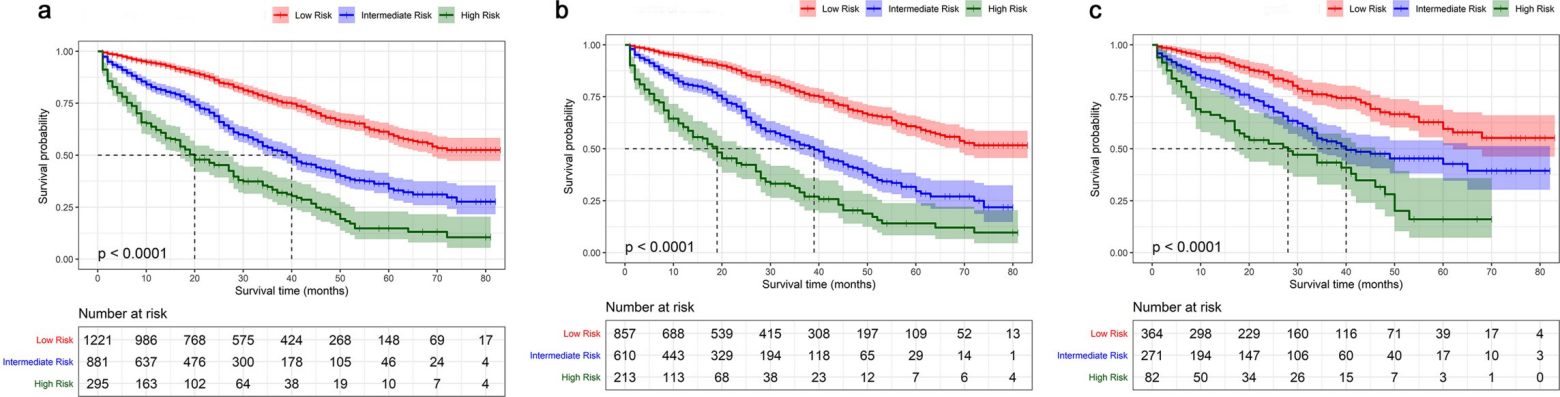
Kaplan–Meier survival curves for low-, intermediate-, high-risk prognostic group in the total cohorts (a), training cohort (b), and validation cohort (c).

Each subgroup of all prognostic variables was given a score relied on the constructed nomogram (Table 3). We calculated personal score for each patient and then gained 2 optimal cut-off values by using X-title. The total population was classified into three prognostic groups, including low-, intermediate-, and high-risk prognostic groups. The number of patients in these three groups was 1221 (50.9%, total points <142), 881 (36.8%, total points from 142 to 208), 295 (12.3%, total points >208), respectively. The median follow-up duration in low-risk group was 27 (IQR, 13–47) months, while 21 (IQR, 8–36) months in intermediate-risk group and 12 (IQR, 4–28) months in high-risk group. KM curves showed the precise differentiation ability for survival probability of diverse prognostic groups (Fig 4). In the total cohorts, 1-year, 3-year, 5-year OS probability for MHBC patients with low-risk prognosis was 94.1%, 76.8%, and 60.1%, respectively, while that for MHBC patients with intermediate-risk prognosis was 81.8%, 52.5%, 33.9%, respectively. For patients in the high-risk prognostic group, their survival prognosis was quite unoptimistic, because their 1-year, 3-year, 5-year OS probability was only 62.1%, 33.4% and 14.8%, respectively.

**Fig 5:**
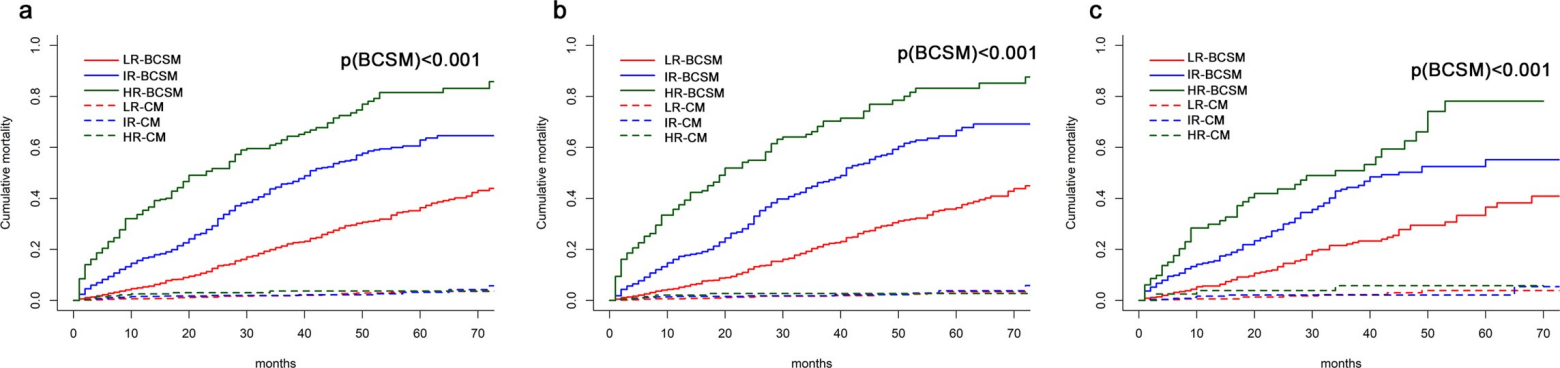
Cumulative mortality curves for low-, intermediate-, high-risk prognostic group in the total cohorts (a), training cohort (b), and validation cohort (c). LR, low-risk; IR, intermediate-risk; HR, high-risk; BCSM, breast cancer-specific mortality, CM, competitive mortality.

Additionally, 1-year, 3-year and 5-year breast cancer-specific mortality of MHBC patients in the low-risk prognostic group was 5.3%, 21.3% and 36.4%, respectively. In contrast, 1-year, 3-year and 5-year breast cancer-specific mortalities of MHBC patients in the intermediate-risk prognostic group was 16.6%, 45.6% and 62.9%, respectively, while that in the high-risk prognostic group was 35.3%, 62.9% and 81.5%, respectively. The CIF curves for breast cancer-specific mortality of patients were shown in Fig 5.

### Benefits from local surgery and chemotherapy

**Fig 6:**
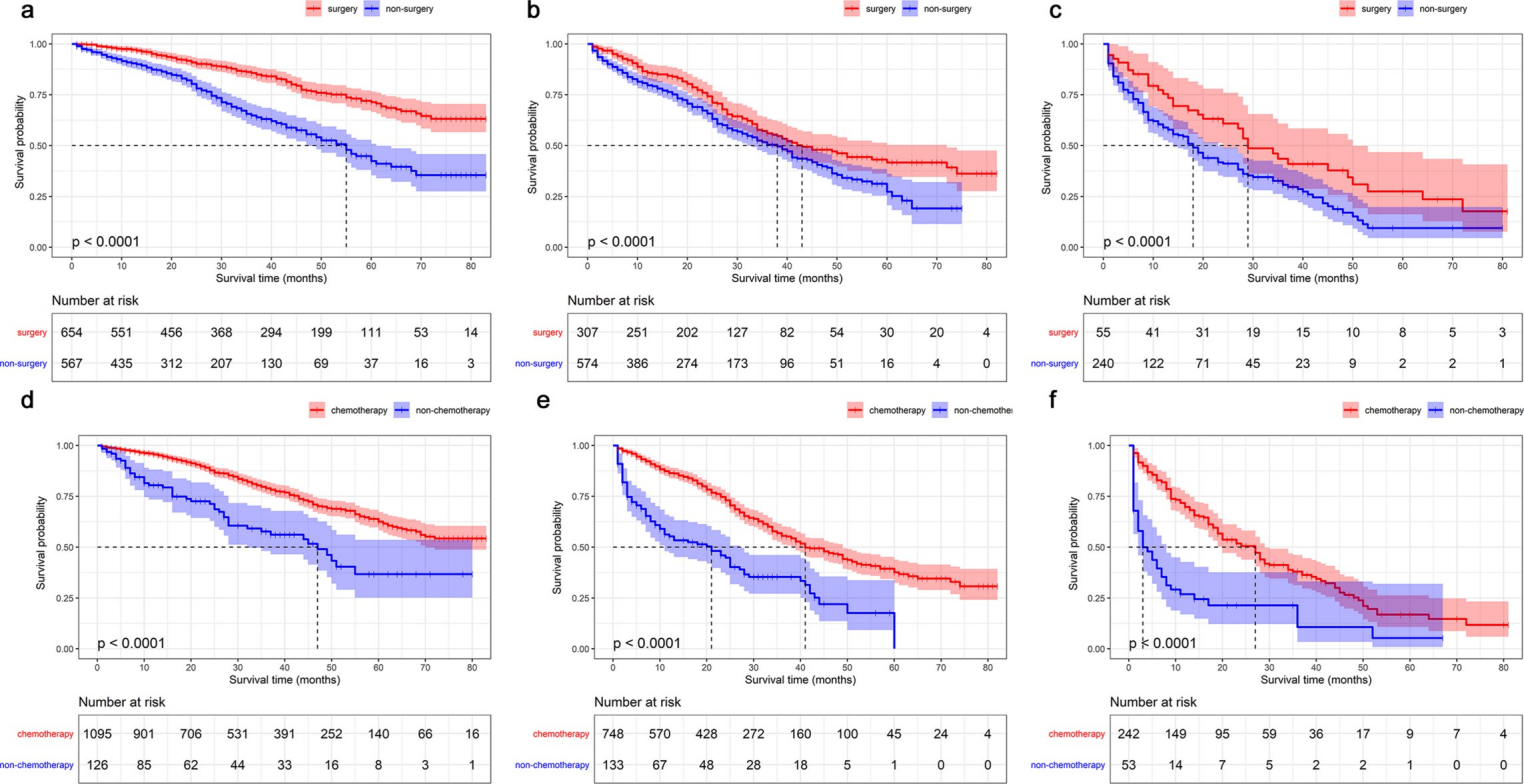
Kaplan–Meier survival curves of metastatic Her2-positive breast cancer patients with or without surgery in (a) low-risk prognostic group, (b) intermediate-risk prognostic group, (c) high-risk prognostic group to evaluate the benefit of overall survival from local surgery. Kaplan–Meier survival curves of metastatic Her2-positive breast cancer patients with or without chemotherapy in (d) low-risk prognostic group, (e) intermediate-risk prognostic group, (f) high-risk prognostic group to evaluate the benefit of overall survival from chemotherapy.

**Fig 7:**
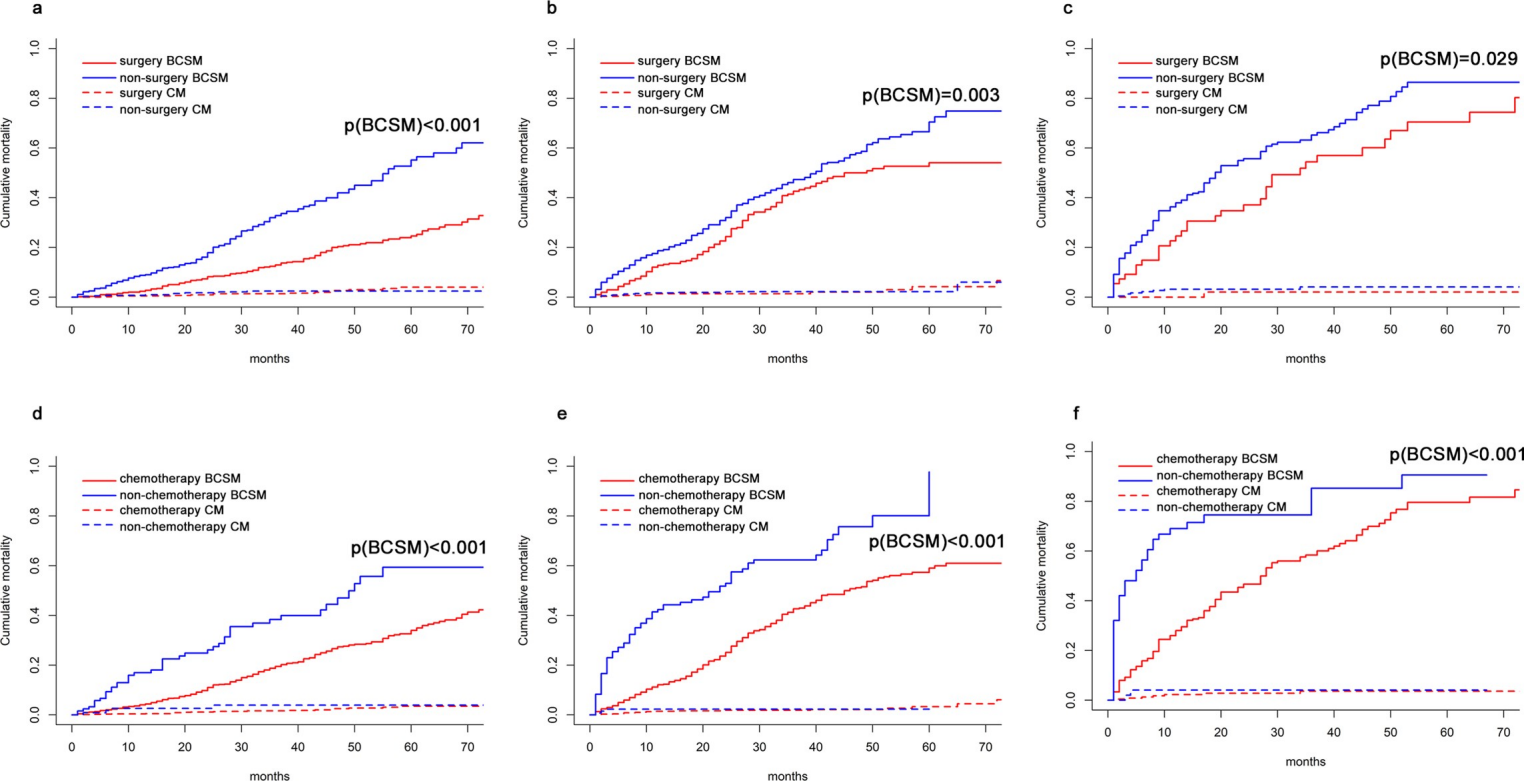
Cumulative mortality curves of metastatic Her2-positive breast cancer patients with or without surgery in (a) low-risk prognostic group, (b) intermediate-risk prognostic group, (c) high-risk prognostic group to evaluate the benefit of breast cancer specific-survival from local surgery. Cumulative mortality curves of metastatic Her2-positive breast cancer patients with or without chemotherapy in (d) low-risk prognostic group, (e) intermediate-risk prognostic group, (f) high-risk prognostic group to evaluate the benefit of breast cancer specific-survival from chemotherapy; BCSM, breast cancer-specific mortality; CM, competitive mortality.

To evaluate benefit from surgery on the primary lesion and chemotherapy for MHBC patients in diverse prognostic groups, we compared the OS probability of patients in different treatment groups. KM curves demonstrated that local surgery significantly prolonged low-, intermediate- and high-risk prognostic MHBC patients’ OS and BCSS duration (Figs 6A–6C and 7A–7C). Especially for patients with low-risk prognosis, local surgery presented with most survival advantage. Local surgery increased a 5-year OS probability by 29.0%, 14.4% and 18.0% for patients in low-, intermediate- and high-risk prognostic group, respectively.

What’s more, all MHBC benefited from chemotherapy (Figs 6D–6F and 7D–7F). Chemotherapy increased a 3-year OS probability by 21.3%, 20.1% and 16.6% for patients in low-, intermediate- and high-risk prognostic group, respectively.

## Discussion

Our research was a sizeable-scale retrospective study, in order to establish a predicted nomogram with risk stratification to predict OS probability for initially diagnosed MHBC patients and provide reference about local treatment for them. Her2-positive presents in 15–20% of BC, which signifies a worse prognosis compared with Her2-negative BC [19]. As a result, several predictive models were constructed to specifically evaluate the prognosis of Her2-positive breast cancer [20]. Luo et al. [21] developed a nomogram to predict survival probability of non-metastatic Her2-positive BC patients. Xiong et al. [22] and Li et al. [8] built nomograms which aimed to quantify the survival probability of MBC patients and the later also found that Her2-positive patients had a worse prognosis compared with other subtypes of MBC patients. In addition, Fujii et al. [12] established a model to predict pathologic complete response (pCR) after neoadjuvant therapy for Her2-positive breast cancer. To our knowledge, this is the first nomogram with risk stratification for predicting OS probability of initially diagnosed MHBC patients.

AJCC 8th edition cancer staging suggests that molecular subtype is an important prognostic factor for BC [14]. In fact, metastatic characteristic of BC is varied from each molecular subtype. Leone et al. [10] demonstrated that HR-/Her2+ malignancy usually had higher odds of visceral metastases, while HR+/Her2+ malignancy was more prone to liver metastases. Wu et al. [13] revealed that liver and brain metastases represented a significantly poorer prognosis for OS and BCSS, whereas bone and lung metastases had no influence on OS prognosis. In our research, the bone was the most common visceral region to suffer from metastasis, while brain metastasis was most infrequent one. Four major visceral metastases all carried out an unfavorable prognosis for MHBC patients, with brain metastasis presenting the poorest prognosis. Meanwhile, ER-positive was a favorable factor for OS prognosis, this finding was consistent with previous research [23, 24].

Her2-positive BC is featured by clinically heterogeneous [25]. Additionally, due to the discrepancies in individual characteristics, the prognosis of patients varied. Our nomogram can be applied widely. On the one hand, it’s suitable for personal prediction, which is of paramount importance for individual therapy and precision medicine. Favorable veracity and discrimination of the model revealed its clinical feasibility. Clinical doctors efficiently assess the OS possibility for patients at their first visits and classify them into the corresponding risk prognostic groups according to their personal total score, to further evaluate benefit from local surgery. Risk stratification has considerable implications for therapeutic cycles and follow-up time arrangement. Higher risk prognosis reveals that an intensive treatment option and a close follow-up period are necessary. On the other hand, our predicted model was suitable to provide reference in prospective trials. Clinicians and investigators estimated prognosis for participants on the basis of their characteristics and then classified them into different experimental groups.

Currently, tumor biology and clinical characteristics influence the therapeutic strategy for MBC patients. According to the recommendation by NCCN guidelines, systemic chemotherapy combined with anti-Her2 therapy is the preferred treatment for MHBC patients [26]. We didn’t include local surgery as a prognostic variable in univariate and multivariate analysis, because local surgery is principally utilized for palliation of symptomatic disease, while the effect for survival prognosis still remains controversial presently [27]. We were supposed to estimate the degree of survival advantage from surgery and chemotherapy for diverse-risk prognostic patients, which was conducive to the individualization in the evaluation of therapeutic efficacy. Chen et al. [28] performed a retrospective study to evaluate the potential benefit of local treatment in 246 stage IV BC patients and found that local surgery or radiotherapy prolonged OS for Her2-positive (3-year, 41.6% vs 8.8%, p = 0.001) and luminal-like (3-year, 66.4% vs 34.4%, p <0.001) BC patients. Recently, Pons-Tostivint et al. [29] conducted a large-scale multicentric retrospective study of 4276 de novo MBC patients, which concluded that locoregional surgery of the primary tumor would improve OS, except for patients with triple-negative molecular subtype. In addition, Zheng et al. [30] draw a conclusion that surgical management of MBC patients carried out a potential survival advantage via reviewing 5173 patients from the SEER database, but the surgical benefit for different subtypes and populations remained unclear. Xiao [31] et al. performed a large-scale meta-analysis consisting of three randomized clinical trials of 714 participants and 30 observational studies of 67,272 participants, and found that resection of primary lesion contributed to better OS (OS (HR 0.65, 95%CI, 0.61–0.70) and distant progression-free survival (HR 0.42, 95%CI, 0.29–0.60), but didn’t contribute to disease-free survival. Theoretically, operative resection of the primary lesion could eliminate the source of newly metastatic focus, alleviate the tumor burden, and potentially reverse the immunosuppression induced by neoplasm [32]. Nevertheless, a prospective phase III trial ABCSG-28 showed that primary focus-surgery didn’t improve prognosis of MBC patients [33]. Bafford et al. [34] proposed that surgery after a diagnosis of MBC couldn’t bring OS benefit on the basis of their retrospective study. Our study recommends implementing primary lesion-surgery for MHBC patients as this would ameliorate their OS and BCSS duration significantly, especially for patients with low-risk prognosis.

Inevitably, there were some limitations in our research. First, information including detailed chemotherapy regimens, Ki-67, multigene status were unavailable from the SEER database. Adding these parameters may further improve predicted power of our model. Second, our research was based on the fact that participants were generally in good performance status. Physical condition is closely related to the tolerance of treatment. Therefore, it’s important to entrench optimal curative pattern according to the general conditions of patients. The optimal opportunity of surgery also remains to be further studied. Additionally, SEER database recorded patients’ information when they were first registered, and metachronous metastasis during follow-up was unclear. Finally, our retrospective research relied on data from the SEER database. Therefore, subsequent external validation in patients from other countries and further large-scale prospective studies are imperative.

## Conclusions

In summary, we constructed a neoteric model to predict 1-year, 3-year, 5-year OS probability for the patients with MHBC diagnosed for the first time and estimate their risk levels of prognosis. Clinicians can design optimal treatment and follow-up protocols according to the risk stratification. Of important, for initially diagnosed MHBC patients who meet surgical requirements and are generally in good physical condition, local surgery could improve survival prognosis, especially for that with low prognostic risk. The optimal opportunity of surgery remains to be further studied.
